# Nature Notes.—X

**Published:** 1912-04-20

**Authors:** 


### THE PRACTITIONER'S RELAXATIONS AND HOBBIES.

### Nature Notes.—X.

**Plovers as Nest Builders—Examples of their Care—A Find—Flint-hidden—A Sitting Plover—Tactics—The Cock Bird—Joint Ruses.**

Plovers prefer a south aspect. They love the sun. They have favourite places, and year by year nest in the same fields and the same part of the field. I have seen it stated that the nest is but a chance depression such as a hoof-mark. Observation of pairs making nests and of many nests had convinced me that a plover's nest is elaborate and constructed with care. The bird takes days to make the nest. Success and satisfaction is often preceded by failure, for it is common to find near a nest others unfinished. As boys we used to call these "false nests." I believe them to be nests begun and abandoned as unsatisfactory. I cannot determine that both birds share in the nest-making. Generally one bird is standing by whilst the other works. I believe the hen bird does all the making. The first stage is to form a slight depression. This is done by carefully scratching out a hole and smoothing it with the breast. The bird stands on the spot selected and throws out earth in small quantities at each scratch of her feet. She turns about, throwing a little earth to this point and that. To mould and smooth he sides of the depression she has scooped she gets down in it and stoops so that her tail is cocked up, and she turns slowly, pressing her breast against the sides of the hollow. The work is not continuous; the processes take some days. The completed hollow is a deep, saucer-shaped depression, the interior diameter of which is about six inches; the upper part of the sides is smooth, but the bottom is lightly covered with small loose fragments of earth, the result of the action of the bird's claws and of the collection of scraps smoothed off the sides. The rim has a slight rampart formed of the earth scratched out.

The next stage is to line the hollow. The lining is commenced by laying material round the upper part of the hollow roughly parallel to the rim. The bottom is at first left bare. The centre part is gradually filled in till a considerable nest is formed. The lining is dry grass, couch-grass chiefly. When the clutch of eggs is completed it numbers four. After the bird has been sitting for a few days the eggs are arranged, or on account of their shape become arranged, so that all the small ends are together in the centre of the nest and the big ends are at a higher level than the small ends. The axis of each egg is at an angle of about 45° to the ground level, and the axes radiate on divergent lines. The shape of the eggs allows or causes this arrangement, which results in economy of space and allows the bird more easily to cover her comparatively large eggs. Each small end fits into a slight depression in the lining material. Any egg can be lifted out and put back without disturbing the others. I have seen the depressions for the small ends so deep as to resemble a shallow thimble. The eggs must remain in exactly the same relative positions during the full time of incubation. The exactness with which the eggs are arranged may explain the care taken in making the nest.

I had an exceptional opportunity of examining a nest a few days ago. There were four eggs. Next day I visited the nest again and lifted the eggs one by one to see the hollows formed by the small ends; they were exceptionally deep. Two days later I saw that the field was being rolled and that there was no hope of successful hatching. As I wished to show nest and eggs to my wife I left them. But when we went to the nest a little later a labourer had already taken the eggs. The roller had not reached the nest, so I carefully examined it and roughly measured it. Over all it was more than six inches. All the lining was dry couch-grass excepting three stalks of withered hard-head and a fragment of dock. That part of the lining that was undermost and nearest the rim seemed to be composed of single pieces, but later additions were bunches of grass with rootlets and pellets of earth on the rootlets. The nest was so firmly woven that I was able to pass my fingers under it and lift it from its hollow without disturbing its shape.

Another nest I pass nearly every day is most elusive. It is close to the line I usually ride going to and from work, but I can never find it except on my way home. It is exceptionally hard to see, because it is on downland that has been cultivated and allowed to go back to grass. Such land is always thickly studded with flints. The nest is amongst the flints in the middle of a scattered heap of horse droppings. As I go to work the bird sees me afar and slips from her nest before I can spot her, and though I have the nest marked to a few yards I cannot find it. But as I ride home I top a rise and see her sitting below me. A plover going to her nest alights some distance from it and runs to it, going cautiously and crouchingly to it, but she often rises directly from her nest if she has been sitting a few days and is suddenly alarmed. If she sees an intruder approaching at a distance I believe she usually runs before taking flight. This though I am not certain about.

The cock bird stays by the nest when the hen is sitting. He keeps a keen look out, and rising gives a cry of warning and flies off to a little distance, sometimes alighting again, sometimes flying to and fro, performing graceful evolutions in the air. Often he may be observed running across the line between the intruder and the nest, watching keenly, now and again pecking at the ground as if feeding, but I verily believe not picking up food, but deceitfully trying to seem unconcerned. After running a little way he gets up, cries his warning, and begins to dart about in the air with loud cries. Whilst the intruder's attention is distracted the hen leaves the nest unobserved. Then the two join protests.

